# Efficient DNA ligation in DNA–RNA hybrid helices by Chlorella virus DNA ligase

**DOI:** 10.1093/nar/gkt1032

**Published:** 2013-11-06

**Authors:** Gregory J. S. Lohman, Yinhua Zhang, Alexander M. Zhelkovsky, Eric J. Cantor, Thomas C. Evans

**Affiliations:** ^1^DNA Enzymes Division, New England BioLabs, Inc., Ipswich, MA 01938-2723, USA, ^2^RNA Biology Division, New England BioLabs, Inc., Ipswich, MA 01938-2723, USA and ^3^Applications Development, New England BioLabs, Inc., Ipswich, MA 01938-2723, USA

## Abstract

Single-stranded DNA molecules (ssDNA) annealed to an RNA splint are notoriously poor substrates for DNA ligases. Herein we report the unexpectedly efficient ligation of RNA-splinted DNA by Chlorella virus DNA ligase (PBCV-1 DNA ligase). PBCV-1 DNA ligase ligated ssDNA splinted by RNA with k_cat_ ≈ 8 x 10^−3^ s^−1^ and K_M_ < 1 nM at 25°C under conditions where T4 DNA ligase produced only 5′-adenylylated DNA with a 20-fold lower k_cat_ and a K_M_ ≈ 300 nM. The rate of ligation increased with addition of Mn^2+^, but was strongly inhibited by concentrations of NaCl >100 mM. Abortive adenylylation was suppressed at low ATP concentrations (<100 µM) and pH >8, leading to increased product yields. The ligation reaction was rapid for a broad range of substrate sequences, but was relatively slower for substrates with a 5′-phosphorylated dC or dG residue on the 3′ side of the ligation junction. Nevertheless, PBCV-1 DNA ligase ligated all sequences tested with 10-fold less enzyme and 15-fold shorter incubation times than required when using T4 DNA ligase. Furthermore, this ligase was used in a ligation-based detection assay system to show increased sensitivity over T4 DNA ligase in the specific detection of a target mRNA.

## INTRODUCTIONS

DNA and RNA ligases catalyse the formation of phosphodiester bonds between 3′-hydroxyls and 5′-phosphates in nucleic acid residues (1–4). DNA ligases operate preferentially on DNA substrates, including single-strand breaks in dsDNA (‘nicked’ substrates) but can, with varying efficiency, also ligate multiple fragments containing complementary overhangs, blunt ends, and nicked substrates featuring mismatched bases or gaps at the ligation junction (5–11). In addition to its ubiquitous use in molecular biology cloning protocols, the ligation reaction has been applied to the detection and profiling of particular nucleotide sequences in the genome through methods such as the Ligase Chain Reaction or amplification of ligated probes via the polymerase chain reaction (PCR) (12–16). The detection of DNA sequences by ligation can improve the sensitivity and specificity for the chosen sequence, particularly in the ability to detect single nucleotide polymorphisms in targets. Likewise, the ligation of single-stranded DNA (ssDNA) probes splinted by RNA followed by detection of ligated product by PCR or quantitative PCR (qPCR) permits the profiling and quantification of RNA species (17–23). This basic methodology has been applied to the detection of specific mRNA transcripts (17–19,24), detection of microRNAs (25,26), mapping of exon connectivity in transcripts (23), detection of single base polymorphisms (21) and unusual RNA modifications (27), and the profiling and quantification of multiple RNA species simultaneously in a given sample (19,23). Detection of ligated products has been accomplished not only by PCR and qPCR, but by methods including rolling circle amplification of circularized DNA probes (25,28), generation of molecular beacon fluorophore or fluorophore-quencher pairs (29), and next-generation sequencing (24).

Robust RNA detection through ligation requires an enzyme able to efficiently ligate ssDNA probe fragments splinted by unbroken complementary RNA strands. The activity of several ligases has been investigated with regard to ligation of nicks in DNA/RNA hybrid substrates (20,21,30–36). T4 DNA ligase is the most commonly used ligase for DNA manipulation and has been reported to ligate ssDNA fragments when splinted by RNA. T4 DNA ligase, however, displays the highest activity on fully DNA substrates and on substrates where the 5′-phosphorylated strand (the ‘donor’) and the complementary strand (the ‘splint’) are DNA, and the polynucleotide providing the 3′-hydroxyl (the ‘acceptor’) is RNA, with only trace activity on RNA-splinted substrates under similar reaction conditions (36). Despite its comparably low activity for these substrates, T4 DNA ligase is the most commonly used enzyme for the ligation of ssDNA splinted by RNA and is the enzyme of choice in the vast majority of published protocols requiring this activity. Protocols have been reported that require excess enzyme, elevated temperature (37°C), and 2–4-h incubation times to accomplish effective ligation of these substrates (21,22). These protocols also require low ATP concentrations (10 µM) to achieve high ligation yields, as it is believed that the primary reaction product of T4 DNA ligase with RNA-splinted DNA substrates is adenylylated donor DNA (AppDNA), a reaction intermediate not normally released into solution in the course of ligation of fully DNA substrates (37,38). If AppDNA is released in solution, it can become a ‘dead end’ product under conditions of millimolar ATP concentrations, as free ligase rapidly reacts with ATP to adenylylate the active site of the enzyme. The adenylylated enzyme cannot bind AppDNA, as the adenylyl group on the enzyme occupies the same binding pocket as the adenylyl group on the AppDNA intermediate (4,39–41). Micromolar ATP concentrations result in a higher steady state concentration of deadenylylated ligase, which can bind and react AppDNA substrates to ligated DNA effectively (37,42).

*Paramecium bursaria* chlorella virus DNA ligase (PBCV-1 DNA ligase) is a small DNA ligase of 298 amino acid residues that has been well characterized as a model ‘minimal’ ligation system (33,40,42–52). PBCV-1 DNA ligase was previously reported to efficiently ligate nicked DNA substrates as well as ligate ssRNA acceptor strands to ssDNA donors using complementary DNA splints, but was found to have no detectable activity for any RNA-splinted substrate (33). Herein we report that, in contrast to these earlier studies, PBCV-1 DNA ligase can carry out the ligation of RNA-splinted DNA substrates with surprising efficiency as compared with other characterized DNA ligases. PBCV-1 DNA ligase displays a faster maximum rate of turnover, a much lower apparent K_M_ and a much higher proportion of direct ligation versus abortive adenylylation than T4 DNA ligase when acting on these substrates. This ligation works across a range of buffer conditions and substrate sequences, and shows little inhibition at 1 mM ATP for most sequences. Further, when tested in a modified RNA annealing, selection and ligation assay (RASL) (23,24), PBCV-1 DNA ligase shortened ligation time and gave significant increase in sensitivity over T4 DNA ligase for detection of defined amounts of luciferase mRNA from a mixture with Jurkat cellular total RNA by qPCR. PBCV-1 DNA ligase’s efficient turnover of, and high affinity for, RNA-splinted DNA substrates shows great potential as a replacement for T4 DNA ligase in molecular biology applications that use these substrates to profile RNA targets.

## MATERIALS AND METHODS

### General

T4 DNA ligase buffer (50 mM Tris pH 7.5 @ 25°C, 10 mM MgCl_2_, 10 mM DTT, 1 mM ATP) was obtained as a 10× stock from New England BioLabs, Inc. (NEB, Ipswich, MA). Other ligation buffers (low Mg, low ATP) were prepared as 10× stocks. Diluent A (10 mM Tris–HCl pH 7.4 @ 25°C, 1 mM DTT, 0.1 mM EDTA, 50% glycerol, 200 µg/ml BSA, 50 mM KCl) was obtained from NEB. Oligonucleotide annealing buffer (10 mM Tris pH 7.5 @ 25°C, 50 mM KCl, 0.1 mM EDTA) was prepared as a 10× stock. High concentration T4 DNA ligase was obtained from NEB; concentration and adenylylation state were determined as previously described (38). HPLC-purified synthetic single-stranded oligonucleotides were obtained from Integrated DNA Technologies as lyophilized solids.

### Preparation of double-stranded oligonucleotide substrates

**Figure 1.**
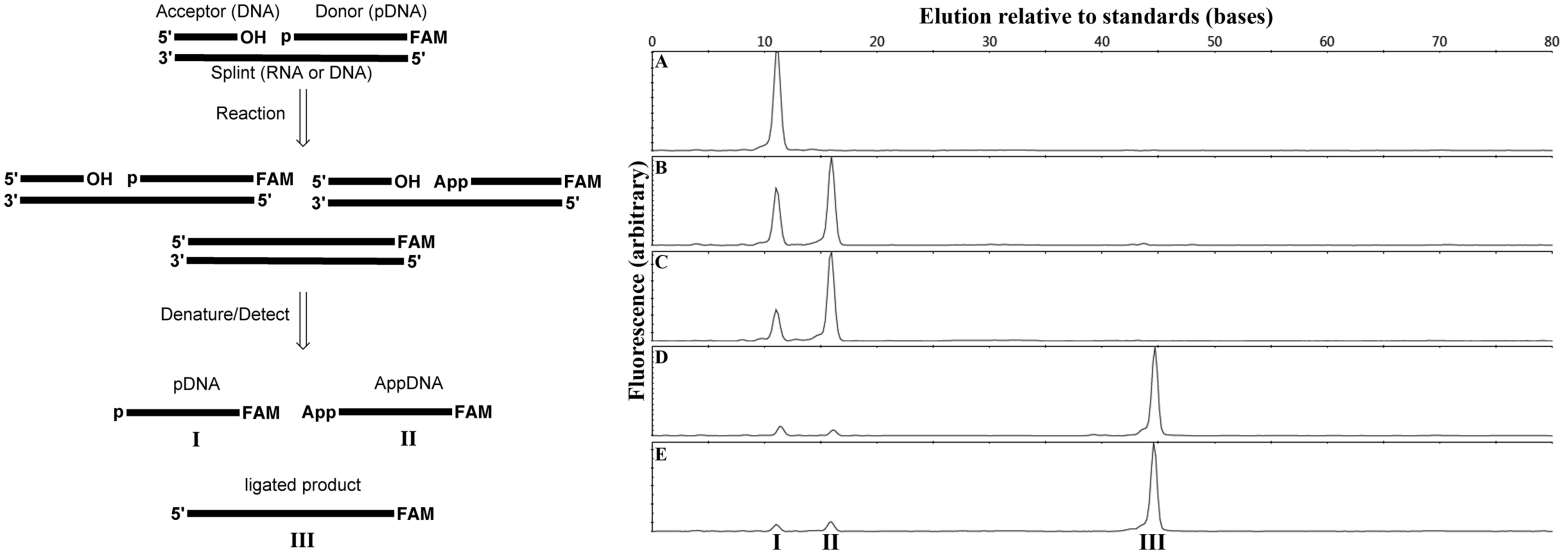
Ligation of DNA splinted by RNA. (Left) Outline of the ligation assay: a 5′-phosphorylated, 3′-FAM labelled DNA ‘donor’ oligonucleotide and an unmodified DNA ‘acceptor’ oligonucleotide are annealed to a complementary RNA or DNA splint. This substrate was reacted with a ligase to form a mixture of unreacted starting material, adenylylated DNA and ligated product. The products were denatured, separated on CE, and detected by fluorescence. (Right) ligation of the standard RNA-splinted substrate in ligase assay buffer for 15 min at 25°C with (**A**) no enzyme, (**B**) 1 µM T4 DNA ligase and 10 µM ATP, (**C**) 1 µM T4 DNA ligase and 1 mM ATP, (**D**) 100 nM PBCV-1 DNA ligase and 10 µM ATP, and (**E**) 100 nM PBCV-1 DNA ligase and 1 mM ATP. Indicated peaks correspond to starting pDNA (I), AppDNA (II) and ligated product (III) as determined by coelution with synthetically prepared standards.

Annealed nicked substrates (Figure 1, left) were composed of an acceptor DNA oligonucleotide TATAACTTTACTTCTAATGN, a 5′-phosphorylated, 3′-6-carboxyfluorescein (FAM)-labelled DNA donor oligonucleotide pNGATGGGACCTACAATGTACCAGAAGCGTC-FAM and an RNA splint of complementary sequence (GACGCUUCUGGUACAUUGUAGGUCCCAUCNNCAAUAGAAGUAAAGUUAUA), where N represents any natural base ((d)A, (d)C, (d)G, or dT(U)) and the RNA splints used contained the correct Watson–Crick base-pairing partners. The FAM-labelled DNA donor (10 µM) was combined with 1.1 molar equivalents each of the DNA acceptor and the DNA or RNA splint in oligonucleotide annealing buffer. This mixture was heated to 85°C for 2 min and allowed to cool slowly in a heat block to room temperature over 3 h. Annealed dsDNA and DNA/RNA hybrid stocks (10 µM in the FAM-labelled fragment) were stored at −20°C. Substrates are identified as N/pN in the text specifying the identity of the 3′-terminal base in the acceptor (dN) and the 5′-phosphorylated base in the donor (pdN), with the dC/pdT sequence being the default sequence used unless otherwise specified. Variant dC/pdT substrates were prepared with a DNA version of the splint, producing a fully DNA nick substrate as previously described (38) or with shorter RNA splints of 10, 20, 30 or 40 bases with the splints distributed evenly around the ligation junction. Two substrates were prepared changing the second base in the donor from a dG to a dT, with a corresponding change in the RNA splint; these substrates both used dN = dC at the acceptor terminus and pdN = dT or dG in the donor, and were designated as dC/pdTdT and dC/pdGdT substrates, respectively. Finally a second nicked substrate, designated Oligo 1 and corresponding to a previously reported RNA-splinted DNA ligation substrate (33), was also prepared by the method described above using an acceptor of sequence CATATCCGTGTCGCCCTT, a donor sequence pATTCCGATAGTGACTACA-FAM and a complementary RNA splint of sequence CUAUCGGAAUAAGGGCGACA.

### Cloning and Purification of PBCV-1 DNA ligase

The wild-type PBCV-1 DNA ligase open reading frame (43) was synthesized by Celtek Genes and subcloned into the Nde I/BamH I sites of the pTWIN1 vector (NEB). The protein was overexpressed in *Escherichia coli* cells (T7 Express lysY/I^q^) grown in Luria-Bertani (LB) medium supplemented with 0.1% glucose and 0.1% MgCl_2_ and induced by Isopropyl β-D-1-thiogalactopyranoside (IPTG) addition (0.4 mM final concentration) at 20°C. Cells were lysed by sonication, and the protein was purified to >95% homogeneity using standard chromatography resins on an ÄKTA Fast protein liquid chromatograph (FPLC). The molecular weight and adenylylation state of the purified protein was confirmed by mass spectrometry as the –Met, fully adenylylated species (Predicted: 34448.3 Da, observed: 34448.9) and the enzyme assayed by standard methods to be free of contaminating DNases and RNases. The storage buffer for the enzyme contained 10 mM Tris–HCl (pH7.4 @25°C), 50 mM KCl, 0.1 mM EDTA, 1 mM DTT and 50% glycerol.

### Ligation assay

Nick ligation assays (Figure 1, left) were performed as previously described (38) in either T4 DNA ligase buffer or a modified buffer when indicated in the text. Ligation of RNA-splinted DNA substrates was typically performed with 10 nM–1 μM T4 DNA ligase or 10 pM–1 μM PBCV-1 DNA ligase at 25°C, unless otherwise indicated. Ligase was pre-incubated in buffer for 2 min at the reaction temperature, then ligation was initiated by addition of substrate (0.5 nM–7.5 µM final concentration, depending on experiment), and mixed by pipetting. Aliquots (5 µl) were removed at the time points indicated, quenched in 50 mM EDTA, 0.1% Triton-X (10 μM), diluted with water so that the total concentration of FAM-labelled molecules was ∼1 nM, and analysed by capillary electrophoresis (CE) fragment analysis on an Applied Biosystems 3730xl Genetic Analyzer (96 capillary array), as previously described (38). For reactions run at <50 nM substrate, the diluted samples were desalted using Edge-Bio PerformaTM VTR 96 well plates before injection. The concentration of each FAM-labelled species was determined as the area of the peak in question divided by the total area of all peaks in the FAM channel, multiplied by the initial concentration of substrate.

### Data analysis

Linear fits to determine initial reaction velocity V_0_ were performed in Microsoft Excel for Mac 2011 (V 14.2.2) by fitting to the first ∼15% of conversion for reactions used to determine Michaelis–Menten parameters, or the first ∼50% of conversion for reactions at 100 nM substrate, which displayed saturating behaviour (linear throughout the time course). Typically the first 20 min of reaction time courses were used for PBCV-1 DNA ligase and up to 5 h for T4 DNA ligases, as deviations from linearity were observed for longer incubation times. Data were typically reported as V_0_ normalized by initial enzyme concentration (V_0_/[E]_0_, units min^−^^1^), and reported values are derived from linear fits to data points that were the average of three repeats. Reported errors are propagated from the uncertainty in the fit slopes and an estimated uncertainty of 5% in the initial enzyme and substrate concentrations. See Supplementary Tables S1–S5 for the results of the fits for each experiment and associated uncertainties and R^2^ values. See Supplementary Figure S1 for plots of representative data sets. Michaelis–Menten parameters were determined by fitting the Michaelis–Menten equation k_cat_ × [S]_0_/(K_M_ + [S]_0_) (where k_cat_ is the turnover number in s^−^^1^, K_M_ is the Michaelis constant in µM, and [S]_0_ is the initial substrate concentration in µM) to a plot of V_0_/[E]_0_ versus [S]_0_, through non-linear regression in KaleidaGraph 4.1.

### qPCR analysis of splint RNA ligation with a mRNA template

A qPCR-detected, modified RASL detection assay (23,24) was performed using T4 DNA ligase or PBCV-1 DNA ligase under identical conditions (Supplementary Figure S6). A pair of probes (probe set A: P5,/5phos/CGGTAAGACCTTTCGGTACTAGATCGGAAGAGCACAC; P3, GGAAGCCTTGGCTTTTGGAACGTTGCGTCGAGTTTTC) was selected for use in a ligation time course study and the target RNA concentration dependence of signal in qPCR. To study the effect of ligation time on qPCR signal, reactions (25 µl) were prepared with 2.5 nM each of the splint probes, 1 or 0.01 ng of luciferase mRNA (Promega, L4561) and 50 ng Jurkat cell total RNA (Life Technologies, AM7858) in T4 DNA ligase buffer. Probes were annealed by incubation at 70°C for 5 min and then cooling to 4°C. PBCV-1 DNA ligase or T4 DNA ligase was added to a final concentration of 500 nM. A control reaction was prepared by addition of 25 µl of a stop solution containing 50 mM EDTA and 10 mM Tris–HCl at pH 7.5 before ligase addition. The ligation reactions were then incubated at 37°C with reactions halted at 15, 30, 60, 120, 240 and 480 min by addition of 25 µl stop solution. The total mRNA and bound DNA was isolated using the NEBNext® Poly(A) mRNA Magnetic Isolation Module (E7490S) with a modified procedure as follows. For each 25 µl reaction 5 µl Oligo d(T)_25_ magnetic beads was aliquoted and washed in batch two times with 2× RNA Binding Buffer. The Binding Buffer was decanted and the beads suspended in a total of 50 µl 2× RNA Binding Buffer. The respective portion of the resuspended beads was then added to each of the stopped ligation reactions. The mix was then heated to 65°C for 5 min, cooled down to 4°C and then incubated at room temperature for 5 min for the mRNA and probe to bind to the Oligo d(T)_25_. The magnetic beads were pelleted using a magnetic rack and washed three times with 50 µl Washing Buffer. To elute the mRNA and probes bound to the Oligo d(T)_25_, 40 µl of Elution Buffer was added to the magnetic beads, heated to 65°C for 5 min followed by transferring the tubes to the magnetic rack. The elution was transferred to a new tube, and 2 µl used for qPCR analysis using iQ™ SYBR® Green Supermix (BioRad, #170-8882) in 25-µl reaction. The cycling conditions started with a single incubation at 94°C for 1 min followed by cycling between 95°C for 10 s and 58°C for 30 s, for 50 cycles.The qPCR primers were Primer5, GTGTGCTCTTCCGATCT, and Primer3, GGAAGCCTTGGCTTTTG. Data for each experiment were reported as the quantification cycle (C_q_), which was inversely proportional to the initial quantity of ligated probe. The signal dependence on mRNA concentration was examined using 2.5 nM of each splint probe and luciferase mRNA from 36.6 ng–36.6 fg in a 25-µl reaction with 2 h ligation time, otherwise identical to above.

## RESULTS

### Rates of PBCV-1 DNA ligase sealing of DNA splinted by RNA under a range of buffer conditions

Initial ligation reactions were performed with 100 nM dC/pdT substrate in either T4 DNA ligase buffer, or a low-ATP version of this buffer (10 µM ATP rather than 1 mM) that was previously reported to facilitate ligation of RNA-splinted DNA by T4 DNA ligase (21,22). Under these reaction conditions, PBCV-1 DNA ligase sealed this substrate rapidly and in high yield while T4 DNA ligase produced only adenylylated DNA product (Figure 1A–E). Reaction with 1 µM T4 DNA ligase for 15 min at 25°C resulted in the detection of exclusively AppDNA at both 10 µM and 1 mM ATP. In contrast, 10-fold less PBCV-1 DNA ligase (100 nM) ligated the substrate to near completion with only small amounts of AppDNA being detected regardless of ATP concentration (Figure 1D and E). To confirm that this unexpected result was not due to an anomalously high activity on the particular substrate sequence chosen, PBCV-1 DNA ligase was reacted with a substrate reported in the original substrate-specificity study (Oligo 1, see ‘Materials and Methods’ section) (33). In our hands, 100 nM Oligo 1 was also efficiently ligated under both high and low ATP conditions in 15 min by 100 nM PBCV-1 DNA ligase (Supplementary Figure S2).

**Figure 2.**
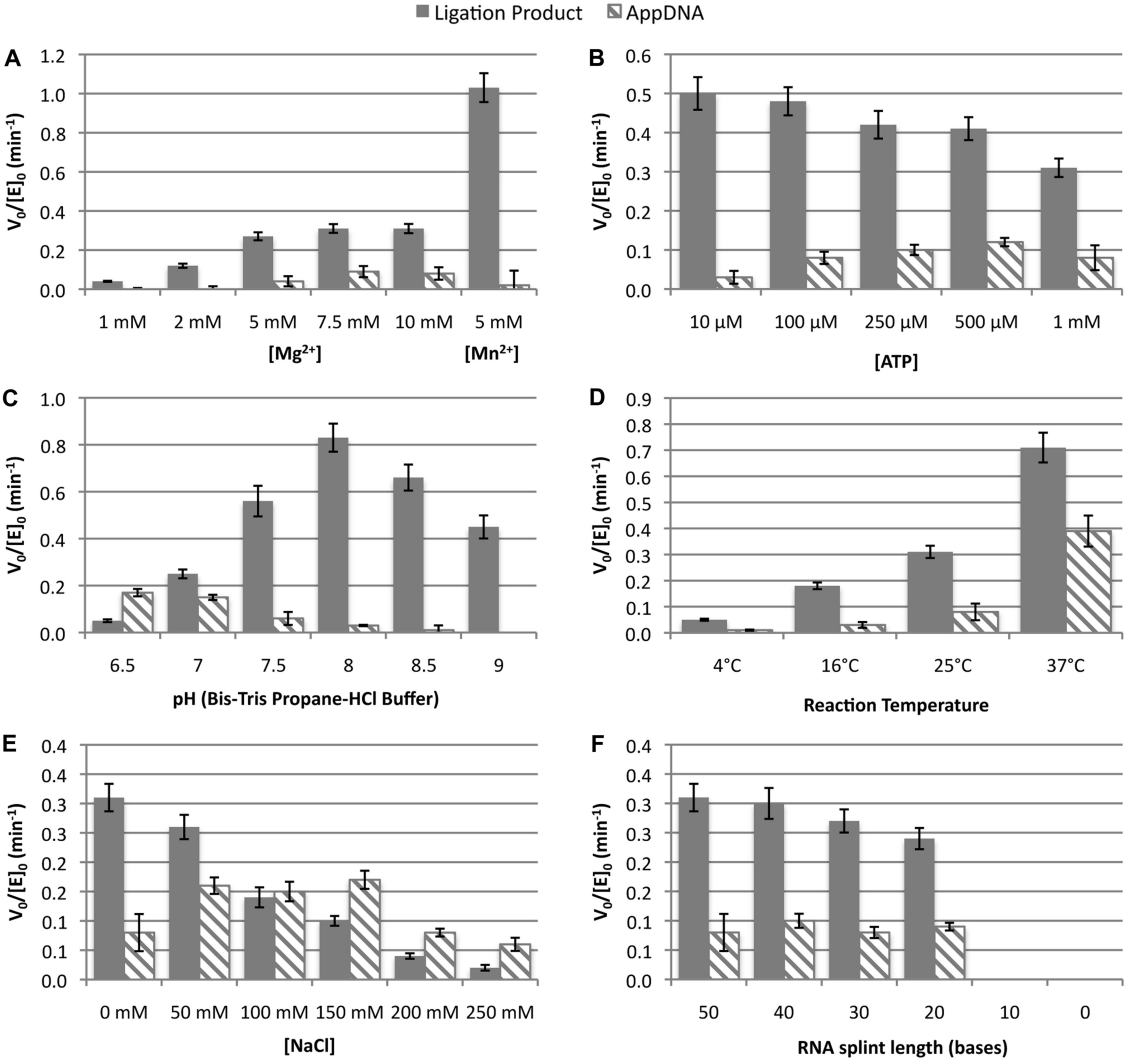
Ligation of RNA-splinted DNA by PBCV-1 DNA ligase under varied reaction conditions. The reaction velocity was measured at 100 nM substrate (dC/pdT) and 10 nM PBCV-1 DNA ligase through linear fits to the first 50% of reaction. The standard reaction conditions used ligase assay buffer (50 mM Tris pH 7.5, 10 mM MgCl_2_, 10 mM DTT and 1 mM ATP) at 25°C. Reactions were run with (**A**) varying MgCl_2_ concentration or substituting MnCl_2_; (**B**) varying ATP concentration; (**C**) varying pH (Bis–Tris propane buffer); (**D**) using standard ligation buffer but varying reaction temperature; (**E**) using standard ligation buffer with added NaCl, and (**F**) under standard reaction conditions but varying the length of the RNA splint. In (F), the splint was distributed evenly around the ligation junction. The solid bars indicate the V_0_/[E]_0_ for formation of ligated product while the hashed bars represent the rate of abortive adenylylation, with the error bars taking into account the uncertainty in the linear fits and initial substrate and enzyme concentrations.

The effect of buffer conditions on the ligation of the dC/pdT substrate by PBCV-1 DNA ligase (Figure 2) was investigated by reaction of 10 nM PBCV-1 DNA ligase with 100 nM dC/pdT substrate (Supplementary Tables S1 and S2). In standard ligation buffer (50 mM Tris pH 7.5, 10 mM DTT, 10 mM MgCl_2_, 1 mM ATP), consumption of substrate obeyed apparent saturation kinetics with a constant V_0_/[E]_0_ = −0.40 ± 0.03 min^−^^1^, but resulted in the formation of both AppDNA and ligated product with V_0_/[E]_0_ of 0.08 ± 0.02 min^−^^1^ and 0.31 ± 0.02 min^−^^1^, respectively (for comparison, under identical conditons T4 DNA ligase produced only AppDNA with a rate of 0.0044 ± 0.0006 min^−^^1^). When the Mg^2+^ concentration was varied (Figure 2A and Supplementary Table S2), it was found that >5 mM MgCl_2_ was needed for maximal activity, as expected (43,52). Mn^2+^ has previously been shown to substitute for Mg^2+^ in the ligation of fully DNA substrates by PBCV-1 DNA ligase with only slight changes to the rate (43). However, when substituting 5 mM Mn^2+^ in place of the Mg^2+^ during the ligation of RNA-splinted DNA, a boost of ∼3-fold in V_0_/[E]_0_ was observed, along with a suppression of AppDNA formation. When the ATP concentration was varied (Figure 2B), little effect on reaction rates was observed except that at the lower (10−100 µM) ATP concentrations, a slight boost in the ligation rate and a notable reduction of AppDNA formation was observed. As described previously for T4 DNA ligase, this effect was most likely due to the conversion of AppDNA to ligated product made possible by the increased fraction of deadenylylated ligase present at 10 µM ATP (21,22). When Bis–Tris propane buffer was used and the buffer pH adjusted across the range of 6.5–9, there was a peak in the reaction rate between pH 7.5 and pH 8. It was also observed that the initial rate of AppDNA formation decreased as pH increased with no detectable rate of AppDNA formation at pH 9. Not surprisingly, the rate of ligation increased as reaction temperature was increased from 4°C to 37°C (Figure 2D), but increased temperatures also resulted in an increased proportion of AppDNA product on initial reaction. However, released AppDNA was also converted to ligated product more rapidly at the higher temperatures (see below), meaning overall reaction time was shortened at 37°C. At 45°C no reaction was observed, but it was unclear whether this result was due to inactivation of the enzyme or melting of the DNA/RNA substrate. These studies were reproduced on a second substrate (C/pG) with very similar trends observed (Supplementary Figure S3 and Supplementary Table S3) despite the preference of this substrate to initially form AppDNA over ligation product (see below). The addition of monovalent cations (NaCl) to the reaction buffer (Figure 2E) strongly inhibited ligation and also drastically increased the fraction of substrate initially converted to AppDNA rather than ligation product. Finally, the reaction was shown to have a minimum RNA splint size, with reaction velocity dropping off slightly as the splint length was reduced from 50 to 20 nucleotides, then dropping to zero for 10 base splints and in the absence of splinting RNA (Figure 2F).

### Kinetic parameters for RNA-splinted DNA substrate consumption

To compare the efficiency of PBCV-1 DNA ligase versus T4 DNA ligase for the ligation of ssDNA splinted by RNA, the Michaelis–Menten parameters were measured for these two enzymes with respect to the consumption of RNA-splinted DNA substrates. Michaelis–Menten parameters for the consumption of starting material were measured for these two enzymes using the standard dC/pdT substrate. The sequence of this oligonucleotide was identical to that used previously to find the Michaelis–Menten parameters at 16°C (k_cat_ = 0.4 ± 0.1 s^−^^1^ and k_cat_/K_M_ = 150 ± 50 μM^−^^1^s^−^^1^) for T4 DNA ligase activity on fully DNA nicked substrates (38). The same sequence was used to determine the apparent Michaelis–Menten parameters for PBCV-1 DNA ligase acting on the fully DNA version of this particular sequence at 25°C under identical buffer conditions. The values determined were k_cat_ = 0.73 ± 0.02 s^−^^1^ and k_cat_/K_M_ = 270 ± 30 µM^−^^1^s^−^^1^ (a K_M_ ∼ 3 nM) (Supplementary Figure S4). These parameters were consistent with the previously reported k_cat_ = 1.1 s^−^^1^ for PBCV-1 DNA ligase with respect to a DNA nick substrate of different primary sequence at 100 nM substrate (51,52) and confirmed that T4 and PBCV-1 DNA ligases are similar in their steady state ligation reaction kinetics for dsDNA nicks.

**Figure 3.**
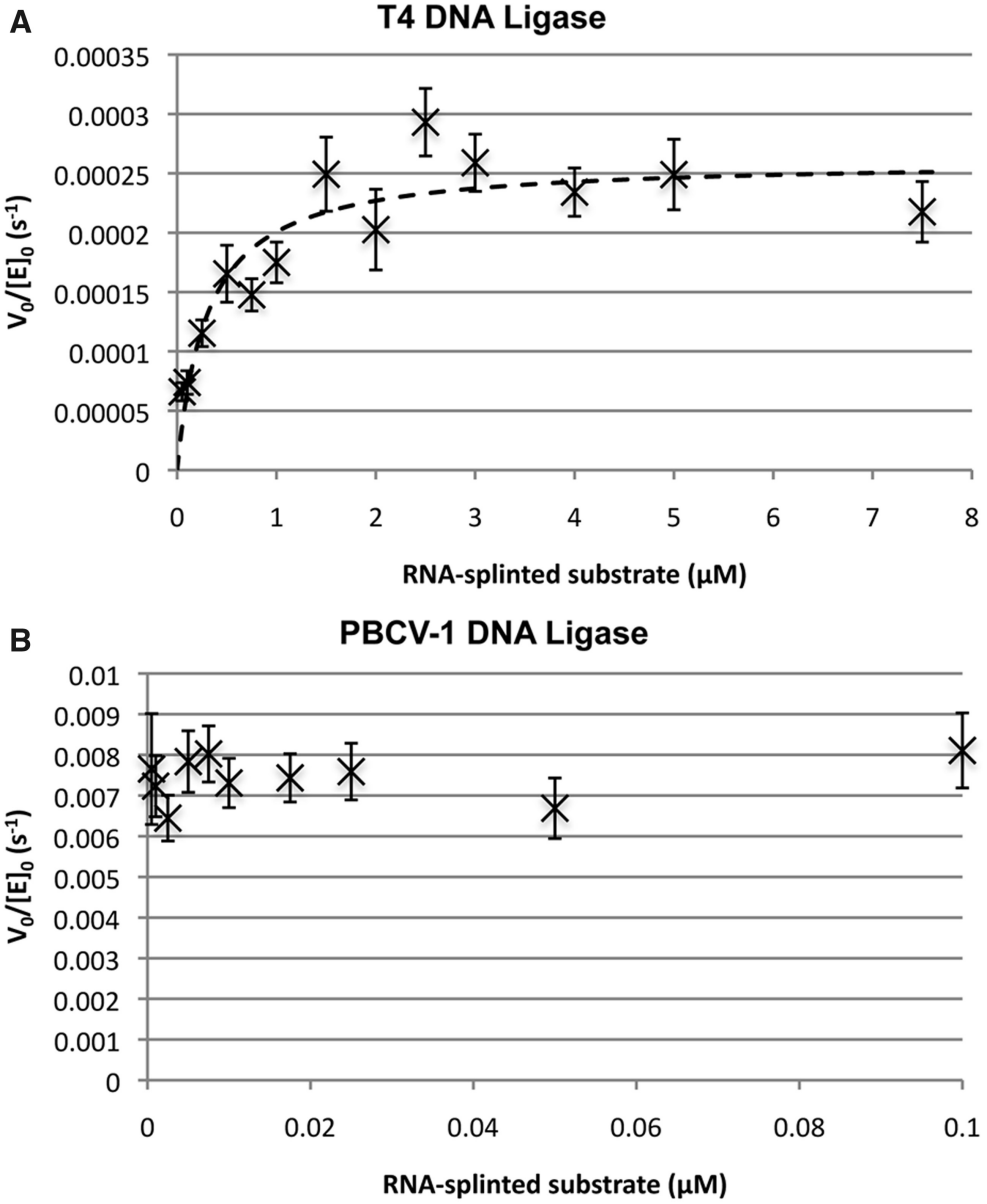
Estimation of Michaelis–Menten parameters for the ligation of DNA splinted by RNA for (**A**) T4 DNA ligase and (**B**) PBCV-1 DNA ligase. Reactions were carried out in ligase assay buffer with 1 mM ATP at 25°C. Initial reaction velocity for the consumption of substrate was measured through fits to the linear region of the reaction (generally the first ∼15% of reaction) with error bars taking into account the uncertainty in the linear fits and initial substrate and enzyme concentrations. Kinetic parameters were determined through fitting the Michaelis–Menten equation to the data by non-linear regression as described in Materials and Methods. For (A), in all reactions the only detected product was AppDNA, and the determined parameters for substrate consumption were k_cat_ = 2.2 ± 0.2 × 10^−4 ^s^−1^ and K_M_ = 300 ± 70 nM. For (B), the reaction products were a ∼ 3:1 mixture of ligated DNA to AppDNA, and the observed V_0_/[E]_0_ was independent of substrate concentration over the range 0.5 nM–100 nM. The approximate k_cat_ is 8 × 10^−3 ^s^−1^ with an upper threshold for the K_M_ estimated to be 1 nM.

In contrast to the ligation of dsDNA nicked substrate, the results for the steady state reaction of RNA-splinted DNA ligation were markedly different for the two enzymes (Figure 3). T4 DNA ligase was reacted with the dC/pdT substrate over the concentration range 50 nm–7.5 µM with at least a 10-fold excess of substrate over enzyme, and the rate of substrate conversion versus time measured at 25°C in standard ligation buffer (Figure 3A and Supplementary Table S4). Under these conditions, the sole product of reaction by T4 DNA ligase was AppDNA at all concentrations and no ligated product was observed. The conversion of substrate to AppDNA did not appear to saturate until >2.5 µM substrate. V_0_/[E]_0_ was determined for each concentration as described in Materials and Methods. Fitting the Michaelis–Menten equation to the plot of V_0_/[E]_0_ versus [S]_0_ gave k_cat_ = 2.2 ± 0.2 × 10^−^^4 ^s^−^^1^ and K_M_ = 300 ± 70 nM for the consumption of substrate and production of AppDNA, with no determinable rate of formation for ligated product under these conditions.

Conversely, PBCV-1 DNA ligase displayed more rapid turnover and much tighter apparent binding (Figure 3B and Supplementary Table S4) for RNA-splinted DNA than was observed for T4 DNA ligase. PBCV-1 DNA ligase was reacted with the dC/pdT substrate in the range 0.5–100 nM in the same buffer at 25°C. The reaction of the RNA splinted DNA substrate formed predominantly ligation product at all concentrations (∼3:1 in favour of ligation product versus AppDNA). The reaction displayed a similar reaction velocity across this range, with an estimated k_cat_ of ∼ 8 × 10^−^^3 ^s^−^^1^ and a K_M_ below the limits of detection for this assay (∼1 nM), representing a 20-fold higher maximum rate of substrate consumption and a K_M_ much lower than that of T4 DNA ligase. It should be noted that the product of the reaction was ∼70% ligated product while T4 DNA ligase produced only AppDNA under these conditions. Thus, the reaction velocity with respect to the formation of ligated product was much greater than 20-fold for PBCV-1 DNA ligase than T4 DNA ligase even under saturating conditions, as the latter must primarily produce product through the reuptake of released AppDNA by trace amounts of deadenylylated enzyme. While the k_cat_ of PBCV-1 DNA ligase for RNA splinted DNA substrates was about 100-fold slower than its k_cat_ for a fully DNA substrate of identical sequence, the apparent K_M_ of PBCV-1 DNA ligase for RNA-splinted substrates was in a similar single-nM range as fully DNA substrates, allowing efficient reaction at low substrate concentration and fairly rapid conversion at high substrate concentration. In contrast the apparent ∼300 nM K_M_ of T4 DNA ligase with respect to RNA-splinted substrates was two orders of magnitude weaker than its affinity for fully DNA substrates.

### PBCV-1 DNA ligase activity on substrates of varied acceptor and donor sequence

**Figure 4.**
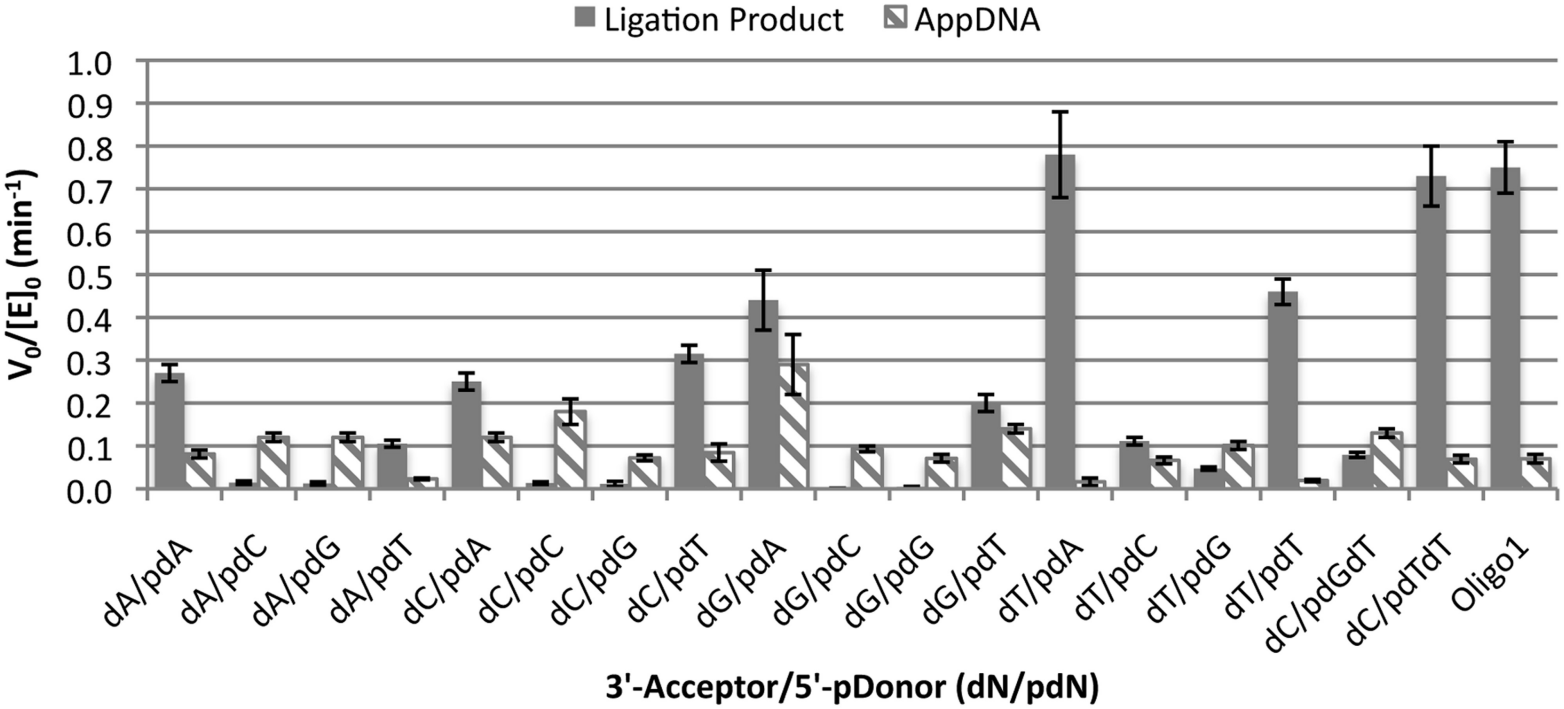
Dependence of ligation rate of RNA-splinted DNA by PBCV-1 on base identity at the ligation junction. The initial rate of reaction for the ligation of DNA splinted by RNA was measured at 100 nM substrate and 10 nM PBCV-1 DNA ligase for a range of substrates varying the base pairs at the ligation junction. All reactions were run in standard assay buffer at 25°C. The solid bars indicate the V_0_/[E]_0_ for formation of ligated product, while the hashed bars represent the rate of abortive adenylylation, with the error bars taking into account the uncertainty in the linear fits and initial substrate and enzyme concentrations. The bases listed on the X axis (dN/pdN) refer to the identity of the base of the DNA acceptor at the ligation junction (dN) and the identity of the phosphorylated base on the donor at the ligation junction (pdN). For all substrates the correct Watson–Crick base-pairing partner was present in the RNA splint.

The V_0_/[E]_0_ dependence on sequence for the ligation of RNA-splinted DNA substrates by PBCV-1 DNA ligase was investigated for all Watson–Crick base pairs at the ligation junction (Figure 4 and Supplementary Table S5). In all cases, the rate of reaction was measured at 100 nM substrate (saturating) and 10 nM PBCV-1 DNA ligase. The sequences tested use the standard substrate sequence outside of the ligation junction as given in the ‘Materials and Methods’ section, with a variable terminal 3′-hydroxyl acceptor (dN) and 5′-phosphorylate donor (pdN) nucleotide at the ligation junction. The V_0_/[E]_0_ for the formation of both ligated product and AppDNA are given (with the total initial rate of consumption of starting material being approximately the sum of these two rates). In all cases, the rate of ligation was linear for at least the first 50% of conversion (typical R^2^ >0.98), indicating that all substrate sequences were in fact present at saturating concentrations in this study. The identity of the donor base had the largest apparent effect on V_0_/[E]_0_, with phosphorylated dA (pdA) and phosphorylated dT (pdT) donors showing fairly rapid formation of ligation product, and pdC and pdG donors showing initial predominant formation of AppDNA and slower overall consumption of starting material. Thus, rapid ligation of RNA-splinted DNA substrate by PBCV-1 DNA ligase appears to be facilitated by a base paired dA or dT residue at the donor side of the ligation junction. This effect was somewhat mitigated if the acceptor base was a dT, as indicated by the dT/pdC and dT/pdG substrates, which show significant initial formation of ligation product (though still slower than all pdA and pdT substrates). In general, a dT on the 5′ (acceptor) side of the ligation junction gave faster ligation rates than any other base, with no clear trend among the other three bases. A substrate with a different sequence, Oligo 1 (which has dA/pdT residues at the ligation junction but different overall sequence), was also tested and found to have ligation rates comparable with the dA/pdT substrate. To test whether the shift in products was due to a weaker K_M_ for the N/pdC and N/pG susbtrates, the assumption of saturation was tested for the dA/pdN series by repeating the reaction at 300-nM substrate. The rates for the dA/pdA and dA/pdT substrates were nearly identical, while the rates for the dA/pdC and dA/pdG decreased somewhat. This result indicates that substrates are largely saturated with rates independent of concentration, but that a possible substrate inhibition effect can reduce the rates of pdC and pdG substrates as initial DNA concentration increases.

As pdC and pdG nucleotides at the donor terminus had such a large effect on ligation V_0_/E_0_, it was considered that the identity of the base at the +2 position of the donor (a dG in the default substrate sequence) might also have a sizeable effect on ligation rate. Thus, two substrates were prepared changing this base to a dT (with the splint base changed to an A), designated dC/pdTdT and dC/pdGdT. These substrates were tested under the same conditions described above, and indeed showed enhanced ligation rates relative to the dC/pdTdG and dC/pdGdG substrates. The dC/pdTdT substrate showed a doubling of the initial ligation rate and a near elimination of observed AppDNA formation. Likewise the dC/pdGdT substrate showed a large increase in the initial rate of formation of ligated product and an overall increase of about 3-fold in the V_0_/[E]_0_ for consumption of substrate as compared with the dC/pdGdG substrate. Thus, it seems that the largest effect on ligation rate and outcomes for RNA-splinted substrates are in the identity of the base-pairs on the donor side of the ligation junction, with dG:C and dC:G base pairs slowing substrate consumption and shifting initial product ratio in favour of AppDNA formation relative to dA:U and dT:A base pairs in these positions.

### Modified reaction conditions allow complete ligation of all substrate sequences by PBCV-1 DNA ligase

**Figure 5.**
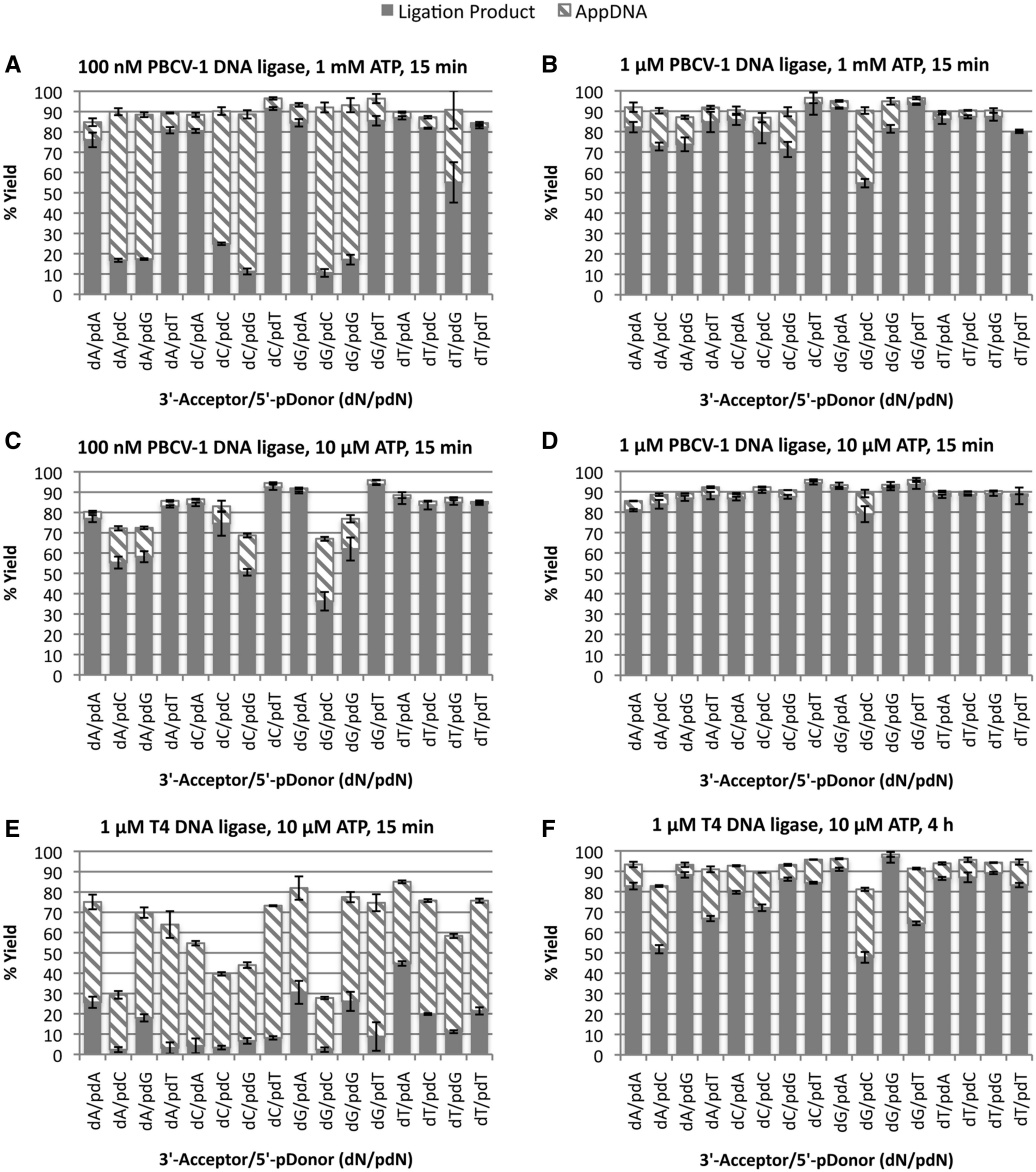
Ligation of RNA-splinted DNA substrates by PBCV-1 and T4 DNA ligases under general reaction conditions. The 16 RNA-splinted DNA substrates, representing all possible base pairs at the ligation junction, were reacted and the extent of ligation and abortive adenylylation measured. The bases listed on the X axis (dN/pdN) refer to the identity of the base of the DNA acceptor at the ligation junction (dN) and the identity of the phosphorylated base on the donor at the ligation junction (pdN). For all substrates, the correct Watson–Crick base-pairing partner was present in the RNA splint. All reactions were incubated at 37°C in 50 mM Tris pH 7.5, 10 mM MgCl_2_, 10 mM DTT, 100 nM RNA-splinted DNA substrate and (**A**) 100 nM PBCV-1 DNA ligase and 1 mM ATP for 15 min; (**B**) 100 nM PBCV-1 DNA ligase and 10 µM ATP for 15 min; (**C**) 1 µM PBCV-1 DNA ligase and 1 mM ATP for 15 min; (**D**) 1 µM PBCV-1 DNA ligase and 10 µM ATP for 15 min; (**E**) 1 µM T4 DNA ligase and 10 µM ATP for 15 min; and (**F**) 1 µM T4 DNA ligase and 10 µM ATP for 4 h. Here the total height of the bar indicates the fraction of starting material converted to products, with the solid portion indicating ligation product yield and the hashed portion indicating AppDNA yield.

While reactions containing PBCV-1 DNA ligase and certain RNA-splinted DNA substrates (dN/pdG and dN/pdC sequences) gave rise predominantly to AppDNA on initial reaction, it was expected that with longer times, excess enzyme or low ATP, conversion of this AppDNA to ligated product could be accomplished for these recalcitrant substrates. Previous reports on the ligation of RNA-splinted DNA using T4 DNA ligase have recommend low concentrations of ATP (10 µM), high concentrations of enzyme (∼1 µM) and 2–4-h incubation at 37°C. (21,22). As PBCV-1 DNA ligase ligates RNA-splinted DNA substrates with a much higher efficiency than T4 DNA ligase (Figure 3), it was expected that shorter incubation times or lower enzyme concentrations would be sufficient to achieve high yields of RNA-splinted DNA ligation for most substrate sequences. A panel of 16 substrates representing all dN/pdN substrates were screened for ligation yield under a variety of conditions (Figure 5). All reactions were run at 37°C to maximize the rate of reaction and the conversion of AppDNA to ligation product. At 100 nM PBCV-1 in standard ligation buffer with 1 mM ATP (Figure 5A), substrates with pdA or pdT donors were ligated to >80% yield in 15 min, though most substrates with 5′-pdG and 5′-pdC donors showed only 10–20% ligation product with the remainder of the yield being AppDNA. Consistent with the results in Figure 4, the dT/pdC and dT/pdG substrates showed a much higher degree of ligation than the other 5′-pdC and 5′-pdG substrates. Reactions with 100 nM PBCV-1 and 10 µM ATP (Figure 5B) showed improved ligation of the 5′-pdC and 5′-pdG substrates, with most substrates ligated to >50% yield in 15 min. Similar results were seen using 1 µM PBCV-1 and 1 mM ATP (Figure 5C), allowing most substrates to be ligated to >70% yields in 15 min. The best ligation extents (to apparent completion) in 15 min were achieved using 1 µM PBCV-1 DNA ligase and 10 µM ATP (Figure 5D). As Mn^2+^ was found to accelerate ligation (see above), reaction conditions were also tested using 5 mM MnCl_2_ in place of MgCl_2_ (Supplementary Figure S5). Indeed Mn^2+^ increased ligation yields of the slower reacting 5′-pdC and 5′-pdG substrates at 100 nM PBCV-1 DNA ligase. Note that in all cases, the maximum ligation yield of these substrates appeared to be 85–90%, with the remaining unreacted material likely representing improperly or incompletely annealed DNA (38,44).

In contrast to PBCV-1 DNA ligase, T4 DNA ligase at 1 µM concentration in buffer containing 10 µM ATP at 37°C (Figure 5E) showed only trace amounts of ligation product for most substrates at 15 min, predominantly producing AppDNA for all sequences. Even the most reactive substrate (dA/pdT) showed only ∼40% conversion. At 4 h under the same conditions (Figure 5F) more complete reaction was observed, similar to the amount of conversion seen with 100 nM PBCV-1 DNA ligase with 10 µM ATP at 15 min (Figure 5B). Therefore, T4 DNA ligase required 10-fold more enzyme and 15-fold longer incubation times to achieve the yields comparable with reactions using PBCV-1 DNA ligase for the ligation of RNA-splinted DNA.

### Performance of PBCV-1 DNA ligase and T4 DNA ligase in a qPCR-detected RASL assay

Ligation of ssDNA probes through an RNA splint has been used previously to detect specific RNA species (21–24). T4 DNA ligase has been typically used in these reactions; however, the studies described herein suggest that PBCV-1 DNA ligase would be effective in these protocols as well. Therefore, we substituted PBCV-1 DNA ligase in place of T4 DNA ligase in a modified RASL assay. DNA ligation products resulting from the modified RASL protocol were detected using qPCR as described in the Materials and Methods. The assay used a defined test system composed of differing amounts of luciferase mRNA mixed with a background of Jurkat cell total RNA. DNA probes were designed such that the two probes would anneal to adjacent sequences in the luciferase mRNA forming an RNA-splinted DNA nick substrate. As described in detail in the Materials and Methods, the RNA template was mixed with the ssDNA probes and annealed by incubation at 70°C followed by cooling to 4°C; DNA ligation was performed at 37°C; the mRNA along with bound probe was purified using poly-dT tagged resin; and finally, ligation products were detected by qPCR.

**Figure 6.**
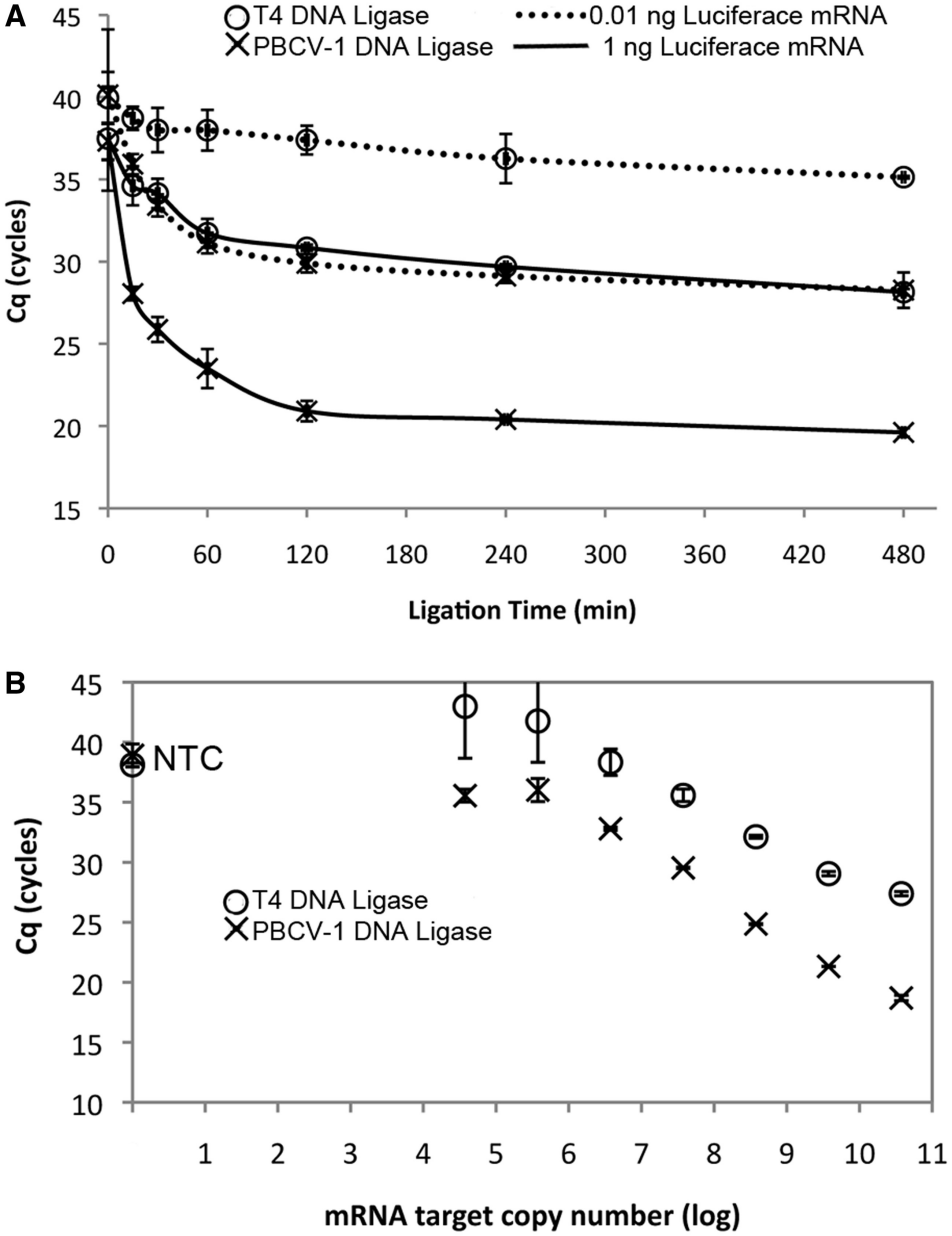
Detection of defined amounts of luciferase mRNA from a mixture with Jurkat total RNA through ligation of specific DNA probes (Probe set A) and detection by qPCR. RNA/DNA probe mixtures were annealed then incubated with either T4 DNA ligase (open circle) or PBCV-1 DNA ligase (×). The qPCR C_q_ for each experiment was recorded, with lower C_q_ indicating higher concentration of ligated probes. (**A**) Ligation time course with either 1 (solid line) or 0.01 ng (dotted line) of luciferase mRNA target, 0–8 h ligation time. (**B**) Dependence of C_q_ after probe ligation with target luciferase mRNA present over a 7 log concentration range using a 2 h ligation time. NTC = no template control. The error bars show one standard deviation of the average of a minimum of three replicates.

To determine the time required for the ligation step to reach completion, a time course using two levels of input luciferase mRNA (1 and 0.01 ng) was performed in which all the steps in the RASL reaction were held constant except for the ligation reaction time (Figure 6A). Both PBCV-1 and T4 DNA ligases generated a reliable signal with 1 ng of input luciferase mRNA. Detectable ligation product accumulated in the first 30 min and reached a >95% maximum signal by 2 h. Interestingly, the amount of ligation products generated in 15 min by PBCV-1 DNA ligase was similar to that produced in 2 h by T4 DNA ligase. The Cq value when the ligation reaction reached a plateau was significantly lower with PBCV-1 DNA ligase than with T4 DNA ligase, 20 and 29, respectively, indicating a larger amount of ligated probe was present in the PBCV-1 DNA ligase-treated sample. Furthermore, at the lower input amount of luciferase mRNA (0.01 ng), only PBCV-1 gave a reliable signal while the T4 DNA ligase reaction gave Cq values that overlapped with that of the no ligation control (Figure 6A); further, the Cq for PBCV-1 DNA ligase and 0.01 ng input mRNA matches the Cq for T4 DNA ligase and 1 ng input DNA throughout the ligation time course, suggesting approximately a 100-fold difference in ligation efficiency. In a separate reaction series in which the ligation step was held constant at 2 h, but the amount of input template was varied, the use of PBCV-1 DNA ligase consistently resulted in lower Cq values for the same input template as compared with T4 DNA ligase (Figure 6B).

## DISCUSSION

We have characterized the ability of PBCV-1 DNA ligase to efficiently ligate ssDNA oligonucleotides splinted by a complementary RNA strand. The ligation of RNA-splinted DNA substrates worked well at a broad range of ATP concentrations (10 µM–1 mM) and across a range of pH with an optimum between pH 7.5 and 8.0. The reaction was enhanced at higher temperatures (up to 37°C), and by substituting Mn^2+^ in place of Mg^2+^ ions. The reaction occurred most effectively with no added NaCl and demonstrated progressively increasing levels of inhibition as the NaCl concentration increased. Ligation was robust for a broad range of substrate sequences, though reaction was slower with a dC or dG in the donor position, particularly when the next downstream base was also a dC or dG. Nevertheless, all sequences tested could be ligated with high yields in much shorter incubation times than required by T4 DNA ligase. Finally, in a proof of principle RASL assay, PBCV-1 DNA ligase demonstrated better performance than T4 DNA ligase showing a consistently lower C_q_ for a given amount of input mRNA target.

These results stand in contrast to the majority of other characterized ligases. For example, *E. coli* (53) and Vaccinia virus DNA ligases (32) are reported to form no detectable ligation product with RNA-splinted DNA substrates. The NAD^+^-dependent ligase from *Melanoplus sanguinipes* (35) is able to ligate these substrates with similar activity to T4 DNA ligase—which, despite being the ligase of choice for this reaction in molecular biology, ligates these substrates poorly with a 1000-fold slower maximal velocity and 100-fold higher K_M_ than for fully DNA substrates. Reaction of RNA-splinted DNA by T4 DNA ligase produces primarily AppDNA on initial reaction, with ligated product appearing only at extended incubation times. Crystal structures of PBCV-1 DNA ligase bound to nicked DNA substrates showed that the substrates were engaged with an RNA-like A-form helix on the acceptor side of the nick, but a DNA-like B-form helix on the donor side (40). Indeed the widespread inability of characterized DNA ligases to form detectable ligation product with fully RNA splints has, along with the crystal structure data, led to the proposal that a strict requirement for a B-form helix on the donor side of the nick was general feature of DNA ligation by DNA ligases (39–41). However, the data in this study show a ∼100-fold depression in the k_cat_, though a similar K_M_ for RNA-splinted DNA ligation versus fully DNA substrate ligation by PBCV-1 DNA ligase. While PBCV-1 still clearly prefers the ligation of fully DNA substrates over hybrid helixes, this rate drop was a relatively minor change in efficiency compared with the ∼10^5^ drop in k_cat_/K_M_ observed for ligation by T4 DNA ligase between these two substrates.

The ∼300 nM K_M_ of T4 DNA ligase for these RNA-splinted substrates suggests much weaker binding of helices containing RNA as compared with fully DNA substrates. The weaker binding may account for the preponderance of AppDNA that accumulated in these reactions—weak binding by virtue of a fast substrate off-rate may mean that the loss of the AppDNA intermediate was much more likely before completion of ligation than in ligation of fully DNA substrates. In the course of ligation of DNA nicks, AppDNA was only observed under single turnover conditions, with steady state ligation reactions showing no observable AppDNA intermediate (38,50,51). This result indicated that product release was only appreciable after phosphodiester bond formation, with the AppDNA reacting on to product much faster than it could dissociate. For the T4 DNA ligase reaction of RNA-splinted substrates, a distorted binding structure could also lead to much weaker binding of the reactive intermediate, making the rate of AppDNA dissociation competitive with a relatively slow rate of phosphodiester bond formation. While the majority of enzyme is re-adenylylated rapidly after dissociation of this intermediate, deadenylylated ligase could rebind this released intermediate for a second chance at ligation. Therefore, most ligation product would be formed from this second binding and reaction event and would be limited by the amount of non-adenylylated ligase in solution. This mechanism would account for the enhanced amount of ligation product formed at low ATP concentrations. For most RNA-splinted substrates, PBCV-1 DNA ligase formed a much higher proportion of ligated product on initial reaction, suggesting that it was able to carry out the phosphodiester bond formation at rates faster than, or at least competitive with, AppDNA intermediate dissociation. Interestingly for the substrates that reacted poorly for PBCV-1 DNA ligase, those with dC or dG bases on the donor at the ligation junction, the shift to AppDNA was not accompanied by an apparent loss of saturation, as the rate of substrate consumption at 100 nM substrate remained linear for at least the first 50% of reaction (and decreased slightly at 300 nM substrate). However the slower k_cat_ of these substrates may simply be just slowed enough relative to the rate of intermediate dissociation, resulting in the shift of product ratios in favour of AppDNA. The higher affinity of PBCV-1 DNA ligase for RNA splinted substrates (as compared with T4 DNA ligase) may additionally allow for the observed faster reaction of free AppDNA to ligated product even at high ATP concentrations. Despite low predicted concentrations of non-adenylylated ligase, higher affinity for the substrate would lead to faster reuptake and conversion to ligated product.

It is possible that the small size of PBCV-1 DNA ligase may explain its ability to accommodate alternative helix forms with greater facility than other DNA ligases. PBCV-1 DNA ligase is one of the smallest characterized ligases, possessing only a nucleotidyl transferase (NT) domain and an oligonucleotide binding (OB) domain, while other DNA ligases generally possess an additional N or C terminal DNA binding domain (DBD) or other domains of uncertain function. Published crystal structures do not exist for many ligases, rendering the positions of these domains when interacting with substrates unknown. In the crystal structure of human DNA ligase 1 bound to DNA, the ligase wraps completely around the bound substrate double helix (39). The nucleotidyl transferase domain binds to the ligation junction, approximately centred on the break, while the DBD and OB domains both make extended contact with the backbone, completely encircling the DNA. In contrast, in the crystal structure of PBCV-1 DNA ligase with a nicked substrate, only a small 29 amino acid residue ‘latch’ domain loops out of the OB domain to contact the DNA where the DBD rests in the human DNA ligase structure (40). The lack of a large additional DBD may give more flexibility in the helix shape accepted by PBCV-1 DNA ligase, making RNA-splinted DNA a more favourable substrate than for the majority of DNA ligases. However, this substrate specificity is not fully relaxed in PBCV-1 DNA ligase, as we observed, consistent with earlier reports, that 5′-phosphorylated RNA was not well tolerated in the donor position (33), and the fully DNA substrate is still the preferred substrate for this enzyme based on k_cat_, by a factor of ∼100.

The high activity of PBCV-1 DNA ligase for ligating ssDNA molecules splinted by RNA has application in methods for RNA detection by ligation (15,16). Such protocols are typically accomplished by annealing DNA probes that are complementary to particular mRNA sequences, mRNA splice junctions, microRNAs or specific RNA modification sites in RNA structures (17–19,21,23–27). Probe sequences can include tags for detection dependent on ligation, for example, reverse molecular beacon fluorophore-quencher or fluorescence resonance energy transfer (FRET) pairs that are brought into proximity only on ligated probes, detection through RCA amplification in a ‘padlock’ protocol, or PCR priming regions allowing detection of ligated DNA probes through amplification (15,16,28). The use of ligation allows probing for specific sequences including single base polymorphisms and splicing variants in RNA. DNA-splinted RNA ligation in methods such as RNA-mediated oligonucleotide Annealing, Selection and Ligation with current high capacity sequencing technologies (RASL-seq) allow for specific quantitation of hundreds of targets in hundreds of samples in parallel and decoding the results via sample sequencing (24). The advantages of substituting PBCV-1 DNA ligase into these protocols are obvious: the high activity and low K_M_ of this ligase compared with T4 DNA ligase permits shortened incubation times and greater sensitivity for detection of low concentrations of target, critical features for modern high throughput molecular biology. Further, the greatly reduced abortive adenylylation potential of PBCV-1 DNA ligase obviates the need for extremely low ATP or use of a coenzyme to react AppDNA side products, allowing detection methods to be run in standard ligation buffers without sacrificing yield or detection efficiency. In a proof of principle RASL-type detection assay, PBCV-1 DNA ligase could be substituted for T4 DNA ligase and resulted in consistently better reaction performance, indicating that further development of PBCV-1 DNA ligase for RNA detection applications is warranted.

## SUPPLEMENTARY DATA

Supplementary Data are available at NAR Online.

## FUNDING

Funding for open access charge: New England Biolabs, Inc.

*Conflict of interest statement*. New England Biolabs, the institution supporting this work, is a manufacturer and vendor of molecular biology reagents, including a variety of DNA ligases.
