# Efficient DNA ligation in DNA-RNA hybrid helices by Chlorella virus DNA ligase

**DOI:** 10.1093/nar/gku792

**Published:** 2014-10-07

**Authors:** G. J. Lohman, Y. Zhang, A. M. Zhelkovsky, E. J. Cantor, T. C. Evans

Nucleic Acids Res. 2014 Feb;42(3):1831-44. doi: 10.1093/nar/gkt1032

The authors would like to apologize for an error in the caption of Figure 5 of their recent manuscript. The data is correctly labeled in the published figure image, but the caption has switched the reaction conditions for panels (B) and (C). The corrected figure caption appears below. This correction does not influence the validity of the results and conclusions of this article.

**Figure 5.**
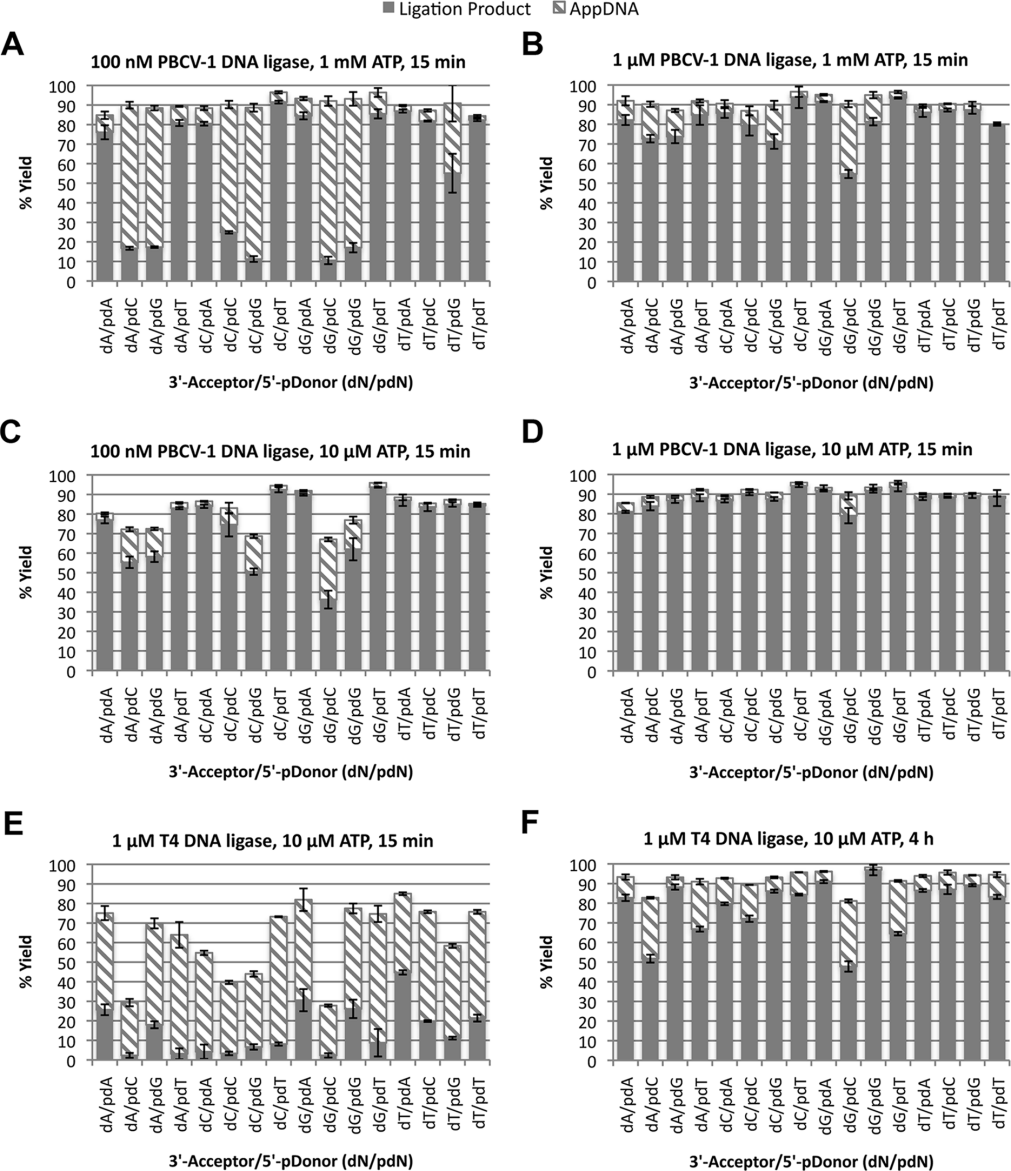
Ligation of RNA-splinted DNA substrates by PBCV-1 and T4 DNA ligases under general reaction conditions. The 16 RNA-splinted DNA substrates, representing all possible base pairs at the ligation junction, were reacted and the extent of ligation and abortive adenylylation measured. The bases listed on the X axis (dN/pdN) refer to the identity of the base of the DNA acceptor at the ligation junction (dN) and the identity of the phosphorylated base on the donor at the ligation junction (pdN). For all substrates, the correct Watson–Crick base-pairing partner was present in the RNA splint. All reactions were incubated at 37°C with 50 mM Tris pH 7.5, 10 mM MgCl_2_, 10 mM DTT, 100 nM RNA-splinted DNA substrate and (**A**) 100 nM PBCV-1 DNA ligase and 1 mM ATP for 15 min; (**B**) 1 μM PBCV-1 DNA ligase and 1 mM ATP for 15 min; (**C**) 100 nM PBCV-1 DNA ligase and 10 μM ATP for 15 min; (**D**) 1 μM PBCV-1 DNA ligase and 10 μM ATP for 15 min; (**E**) 1 μM T4 DNA ligase and 10 μM ATP for 15 min; and (**F**) 1 μM T4 DNA ligase and 10 μM ATP for 4 h. Here the total height of the bar indicates the fraction of starting material converted to products, with the solid portion indicating ligation product yield and the hashed portion indicating AppDNA yield.

